# Influence of Model Design and Printing Orientation on the Dimensional Accuracy of 3D-Printed Models for Implant-Supported Restorations

**DOI:** 10.3390/ma19030516

**Published:** 2026-01-28

**Authors:** Felix Förtsch, Antonius Klemt, Valentin Kabst, Harald Schwandner, Manfred Wichmann, Ragai Edward Matta

**Affiliations:** Department of Prosthodontics, University Hospital Erlangen, Glückstrasse 11, 91054 Erlangen, Germany; felix.foertsch@uk-erlangen.de (F.F.); antonius.klemt@uk-erlangen.de (A.K.); valentin.kabst@uk-erlangen.de (V.K.); harald.schwander@uk-erlangen.de (H.S.); claudia.ehrhardt@uk-erlangen.de (M.W.)

**Keywords:** additive manufacturing, 3D printing, digital light processing, printing orientation, dimensional accuracy, implant-supported restorations

## Abstract

Dimensional accuracy of 3D-printed implant models is essential for precise implant-supported restorations. The objective of this study was to evaluate the influence of printing orientation and model base design on the accuracy of implant position transfer. A standardized maxillary model with four implants was scanned using an intraoral scanner. Solid and hollow models were designed and printed using digital light processing (DLP) technology at orientations of 0°, 45°, and 90° (*n* = 10 per group). All models were digitized with a high-precision industrial scanner, and implant position deviations were determined by comparing corresponding reference points with the master model. Data were analyzed using two-way analysis of variance and post hoc tests (α = 0.05). Printing orientation significantly affected accuracy (*p* < 0.001). Models printed at 45° showed the highest deviations, whereas those printed at 0° and 90° exhibited comparable and superior accuracy. Model design (solid vs. hollow) had no significant influence at 0° and 90°, but hollow models were more accurate at 45° (*p* < 0.001). Mean deviations ranged from 131 μm to 382 μm. Printing at 0° or 90° is recommended, while 45° orientations should be avoided. Model design showed minimal effect on accuracy.

## 1. Introduction

Dental implants have become a well-established and highly effective treatment modality for the replacement of missing teeth [1]. They enable the restoration of function, aesthetics, and quality of life in patients with partial or complete tooth loss, thereby assuming a central role in modern restorative dentistry [2,3]. The long-term success of implant-supported restorations depends on the precise transfer of the intraoral implant position to the dental laboratory, ensuring an accurately fitting superstructure [4].

Implant therapy has traditionally been based on conventional procedures, including impression taking using elastomeric materials, the fabrication of stone casts, and restoration manufacturing using the lost-wax technique [5,6]. Advances in digital technology now enable a fully integrated digital workflow. In this approach, intraoral scanning replaces conventional impression techniques, restorations are virtually designed using a computer-aided design software, and subsequently fabricated through computer-assisted subtractive or additive manufacturing processes [7,8].

Despite the ongoing digitalization of dental workflows, the fabrication of physical models remains essential in laboratory practice. Such models are indispensable for verifying fit, occlusion, interproximal contacts, and the passive adaptation of multi-unit superstructures [9]. Even minor deviations in the digital transfer can lead to clinically relevant discrepancies, making the dimensional accuracy of printed models a critical factor for treatment success [10].

Among additive manufacturing techniques, stereolithography (SLA) and digital light processing (DLP) are most widely used in dentistry [11]. Both technologies polymerize liquid resins layer by layer, but differ in their exposure principles: SLA employs a laser to trace each layer point by point, whereas DLP cures entire layers simultaneously through digital projection [12]. Due to their high accuracy and shorter production times, DLP systems are particularly suitable for dental model fabrication and chairside applications [13,14].

In addition to the printing technology itself, several process parameters influence the precision of additively manufactured models. The internal structure of the model affects both material consumption and dimensional stability [12]. Furthermore, the printing orientation relative to the build platform significantly impacts trueness and precision. The most frequently investigated orientations in the literature are 0°, 45°, and 90°, with most studies reporting the highest accuracy at a 0° orientation [15]. However, current evidence remains inconsistent and depends on factors such as printing technology, material properties, and model geometry. Conflicting findings regarding optimal wall thickness and internal design further hinder the establishment of standardized recommendations [15,16,17].

While the accuracy of models used for diagnostic or tooth-supported restorations has been extensively validated and is generally within clinically acceptable limits, implant-prosthetic models impose far higher demands on dimensional precision—particularly concerning the spatial reproduction of implant positions [17,18]. To date, only two comparative studies have investigated the influence of model design and printing orientation on the accuracy of printed implant models, indicating a clear need for further research in this area [19,20].

Therefore, the present study aimed to investigate the combined influence of printing orientation and model base design on the dimensional accuracy of additively manufactured implant models. The null hypothesis stated that neither printing orientation nor model base design would significantly affect the accuracy of the implant models.

## 2. Materials and Methods

A standardized maxillary implant model fabricated from type IV dental stone was used for this study. The model included four bone-level implants (Straumann^®^ BLX, Ø 3.8 mm × 12 mm; Straumann GmbH, Freiburg, Germany) positioned in the first quadrant at sites 12, 14, 15, and 17, while the second quadrant was fully dentate. Implants at sites 12, 14, and 15 were inserted perpendicular to the model base, whereas the implant at site 17 was placed with a 25° distal angulation. The master model was digitized using an intraoral scanner (Primescan, Dentsply Sirona, Bensheim, Germany) in combination with scanbodies (LX 1400-SB, Medentika GmbH, Hügelsheim, Germany). The scanbodies were hand-tightened, and all scans were performed by an experienced clinician in accordance with the manufacturer’s recommendations. Based on the obtained scan data, both solid and hollow digital models were designed using exocad software (DentalCAD 3.2, Exocad GmbH, Darmstadt, Germany). The design parameters are summarized in Table 1.

**Figure 1:**
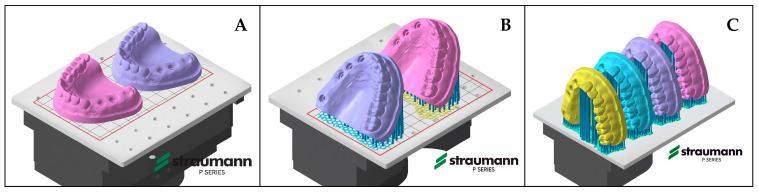
Models prepared for printing. (**A**) 0°, two models; (**B**) 45°, two models; (**C**) 90°, four models.

**Figure 2:**
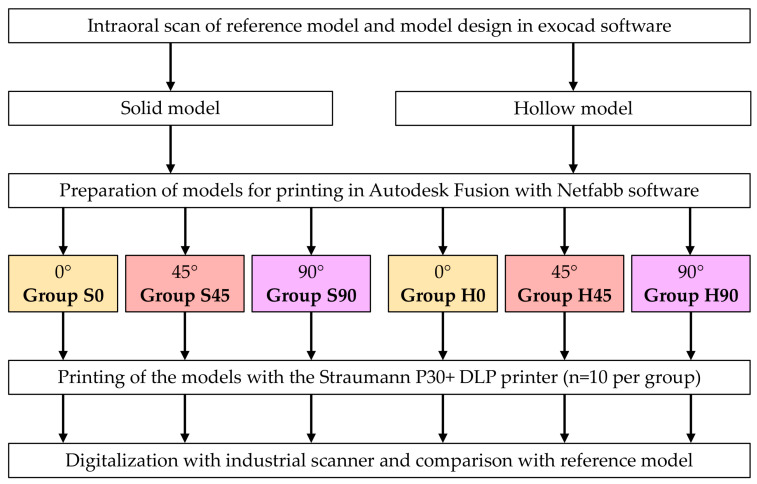
Overview of the study design and study groups.

To evaluate the influence of different printing orientations, the solid and hollow models were prepared for 3D printing at printing orientations of 0°, 45°, and 90° using Autodesk Fusion with Netfabb software (Autodesk Netfabb Basic 2022.0, Autodesk Inc., San Francisco, CA, USA) (Figure 1). The combination of model type and printing orientation resulted in six experimental groups: S0 (solid model, 0° printing angle), S45 (solid model, 45° printing angle), S90 (solid model, 90° printing angle), H0 (hollow model, 0° printing angle), H45 (hollow model, 45° printing angle) and S90 (solid model, 90° printing angle) (Figure 2).

For each group, ten models were printed from a photopolymer resin (Sheraprint model desert, SHERA Werkstoff-Technologie GmbH, Lemförde, Germany) using a DLP printer (Straumann P30+, Straumann GmbH) in accordance with the manufacturer’s recommendations. The following parameters were used during the printing process: a layer thickness of 50 µm, an exposure time of 1.2 s per layer, and support densities of 2 mm on all surfaces and 0.7 mm along all edges. After cleaning of the models (Straumann P wash, Straumann GmbH), support structures were removed, and model analogs (LX 80, Medentika GmbH) were inserted using the corresponding placement instrument (LX 80 T, Medentika GmbH) following the manufacturer’s guidelines. Subsequently, all models were post-cured under vacuum as recommended by the manufacturer (Straumann P cure, Straumann GmbH).

**Figure 3:**
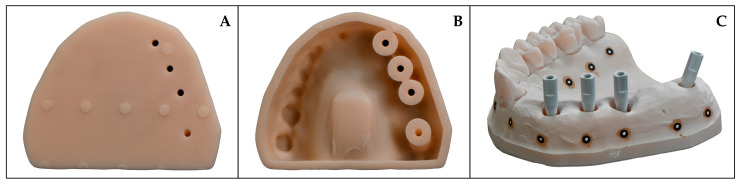
(**A**) Solid model, (**B**) hollow model, (**C**) model prepared for scanning with industrial scanner.

The reference model and all printed models were digitized using an industrial high-precision scanner exhibiting an accuracy of 4 μm (ATOS Triple Scan, Carl Zeiss GOM Metrology GmbH, Braunschweig, Germany). Reference markers were attached to the model surfaces, which were subsequently coated with a thin layer of titanium powder (Rutile Titanium White, GOM GmbH, Braunschweig, Germany) to reduce light reflection during scanning (Figure 3).

**Figure 4:**
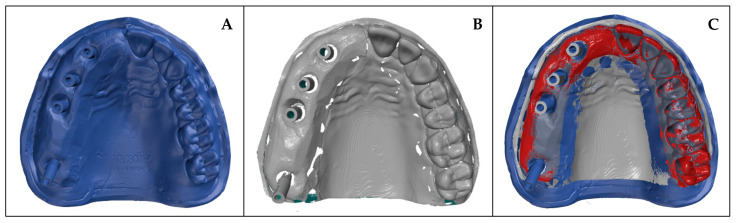
(**A**) Digitized reference model, (**B**) digitized 3D-printed model, (**C**) best-fit matching, red indicates the region used for superimposition.

The subsequent data evaluation was performed using the software ATOS Professional 2020 (Carl Zeiss GOM Metrology GmbH). All digitized 3D-printed models were initially automatically superimposed with the reference scan. In a second step, a best-fit matching was carried out over the alveolar ridge while explicitly excluding the scanbodies (Figure 4). This approach was chosen to avoid potential underestimation of implant position deviations that could occur if the alignment were based on the scanbody surfaces.

For implant position comparison, identical and reproducible reference points were defined at the center of each coronal scanbody surface using best-fit cylinders and planes. The spatial deviation was determined by comparing the corresponding scanbody reference points between the virtual reference model and the digitized 3D-printed models. For each model, the mean deviation of the four individual scanbodies was calculated and used for statistical analysis.

Statistical analysis was performed using the R program (version 4.5.2, R Core Team, R Foundation for Statistical Computing, Vienna, Austria). Initially, a two-way analysis of variance (ANOVA) was performed to investigate the effects of the two examined parameters and their potential interaction. Subsequently, the individual study groups were compared using post hoc tests with Holm correction. The level of significance was set at 0.05.

## 3. Results

In the two-way ANOVA, printing angle had a significant effect (*p* < 0.001), whereas model design showed no significant effect (*p* = 0.154). Examination of the interaction revealed a statistically significant interaction between the two variables (*p* < 0.001). Therefore, post hoc tests were performed to compare the individual study groups conditional on the other factor.

Analysis of the spatial deviations revealed significantly higher deviations in the models printed at a 45° printing angle compared with those printed at 0° and 90°. The largest difference in mean deviation was observed between groups S45 and S90, amounting to 251 μm, whereas the smallest difference was found between groups H45 and H90, with 76 μm. No significant difference was detected between the 0° and 90° printing angles (Table 2 and Table 3).

**Figure 5:**
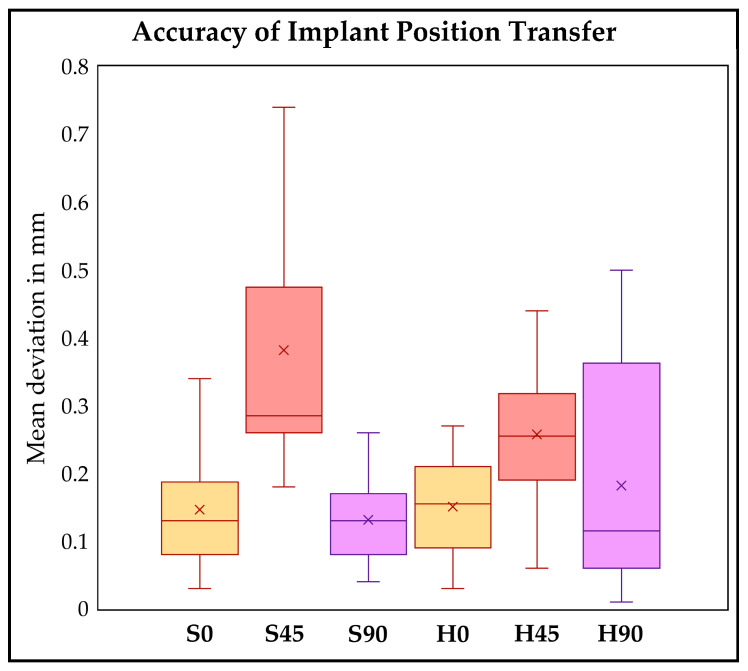
Box-whisker-plot showing the mean deviation in mm for all study groups.

When comparing the two model designs, no significant differences were observed at printing angles of 0° and 90°. However, for the models printed at 45°, the hollow models demonstrated significantly higher accuracy, with a mean deviation 124 μm lower than that of the corresponding solid models (Table 2 and Table 3). Figure 5 presents a graphical representation of these results in the form of a box-and-whisker plot.

## 4. Discussion

The present study investigated the influence of printing orientation and model base design on the dimensional accuracy of additively manufactured implant models. The results demonstrated a statistically significant effect of printing orientation, whereas the internal model design (solid vs. hollow) showed no significant influence on accuracy. Specifically, models printed at 0° and 90° exhibited superior trueness compared with those printed at 45°, with no significant difference between the two favorable orientations. Consequently, the null hypothesis must be partially rejected, as printing orientation—but not model base design—had a significant impact on the accuracy of implant position transfer.

These findings contribute to the ongoing discussion on the optimal printing orientation for dental model fabrication. A recent systematic review by Alghauli et al. synthesized evidence from 27 studies and reported the highest accuracy for models printed at 0°, whereas 90° orientations were associated with the greatest deviations. Notably, the majority of included studies focused on fully dentate models, typically fabricated for orthodontic or aligner applications, which impose less stringent accuracy demands than implant-prosthetic models. Furthermore, Alghauli et al. found no consistent differences between solid and hollow designs, a finding that is in full agreement with the results of the present investigation [15]. The alignment of these results reinforces the assumption that, under standard printing conditions, the internal architecture of a model exerts minimal influence on global dimensional fidelity.

In contrast, the evidence regarding implant-supported models remains scarce. To date, only two comparative studies have specifically examined the impact of printing orientation on the accuracy of implant analog positions in 3D-printed models. García et al. reported the highest trueness and precision at 45° orientations [19], while another pilot investigation using similar DLP technology also identified 45° as the most accurate configuration [20]. These findings contradict both the outcomes of the current study and the conclusions of the systematic review by Alghauli et al. [15]. Several methodological factors may explain this discrepancy: differences in printer technology, resin composition, layer thickness, and post-curing protocols can all substantially affect polymerization shrinkage and layer-to-layer stability. Moreover, variations in implant geometry, number of analogs, and alignment strategies during data analysis may contribute to divergent results.

The contradictory findings among available studies highlight that no consensus has yet been reached regarding the optimal printing orientation for implant models. While the present data suggest that 0° and 90° yield clinically acceptable accuracy, further comparative studies using standardized implant configurations and different printing systems are required. Expanding the current evidence base will be essential to establish reliable parameters for the fabrication of precise 3D-printed implant models.

Nevertheless, several limitations of the present investigation must be considered when interpreting these findings. The study was conducted under standardized in vitro conditions, which do not fully replicate the complex intraoral environment encountered in clinical practice. Factors such as saliva, limited mouth opening and variable lighting conditions can substantially influence the performance of intraoral scanners and thus the accuracy of digital impressions. Consequently, the trueness values obtained in this study may represent an idealized scenario that could differ under real intraoral circumstances. Furthermore, only one DLP printing system was evaluated, limiting the generalizability of the results, as different devices, materials, and calibration protocols may exhibit variable accuracy and polymerization behavior. Additionally, the study focused exclusively on a single maxillary implant configuration with four implants in a partially edentulous model. Other implant numbers, angulations, or inter-implant distances may produce different outcomes. Therefore, future research should include clinical investigations and a broader range of scanning and printing systems and diverse implant scenarios to validate the transferability of these findings to real-world clinical applications.

## 5. Conclusions

Taking into account the limitations of this in vitro study, the results support the following conclusions: Printing orientation has a significant impact on the dimensional accuracy of implant position transfer in DLP-manufactured models. In particular, a printing angle of 45° should be avoided, as orientations of 0° and 90° demonstrated more favorable accuracy outcomes. By contrast, model design did not significantly influence accuracy under the conditions investigated. From a practical standpoint, the evaluated hollow design may, therefore, be preferred to improve material efficiency without compromising clinically relevant precision.

## Figures and Tables

**Table 1:** Design parameters for the construction of the models in the exocad software.

General parameters	Horizontal distance between model analogs	50 μm
	Thickness of implant reinforcement	1 mm
	Base height	3 mm
Parameters of Hollow Model	Wall thickness	2.5 mm
	Base threshold	1 mm
	Hollow cavity diameter	2 mm

**Table 2:** Descriptive statistics of the spatial deviation of the implant position for all study groups: Mean deviation (Mean) with standard deviation (SD) and the minimum (Min) and maximum (Max) deviation in μm.

Group	Mean [μm]	SD [μm]	Min [μm]	Max [μm]
S0	147	84	30	410
S45	382	206	180	910
S90	131	58	40	260
H0	151	66	30	270
H45	258	94	60	440
H90	182	164	10	500

**Table 3:** *p*-values for all statistical comparisons.

Comparison	*p*-Value
S0 vs. H0	0.879
S45 vs. H45	<0.001
S90 vs. H90	0.068
S0 vs. S45	<0.001
S0 vs. S90	0.578
S45 vs. S90	<0.001
H0 vs. H45	<0.001
H0 vs. H90	0.263
H45 vs. H90	0.014

## Data Availability

The original contributions presented in this study are included in the article. Further inquiries can be directed to the corresponding author.

## References

[1] Bassir S.H., El Kholy K., Chen C.Y., Lee K.H., Intini G. (2019). Outcome of early dental implant placement versus other dental implant placement protocols: A systematic review and meta-analysis. J. Periodontol..

[2] De Boever A.L., Quirynen M., Coucke W., Theuniers G., De Boever J.A. (2009). Clinical and radiographic study of implant treatment outcome in periodontally susceptible and non-susceptible patients: A prospective long-term study. Clin. Oral Implants Res..

[3] Duong H.Y., Roccuzzo A., Stähli A., Salvi G.E., Lang N.P., Sculean A. (2022). Oral health-related quality of life of patients rehabilitated with fixed and removable implant-supported dental prostheses. Periodontology 2000.

[4] Flügge T., van der Meer W.J., Gonzalez B.G., Vach K., Wismeijer D., Wang P. (2018). The accuracy of different dental impression techniques for implant-supported dental prostheses: A systematic review and meta-analysis. Clin. Oral Implants Res..

[5] Keith S.E., Miller B.H., Woody R.D., Higginbottom F.L. (1999). Marginal discrepancy of screw-retained and cemented metal-ceramic crowns on implant abutments. Int. J. Oral. Maxillofac. Implants.

[6] Vigolo P., Givani A. (2000). Clinical evaluation of single-tooth mini-implant restorations: A five-year retrospective study. J. Prosthet. Dent..

[7] Negreiros W.M., Hamilton A., Gallucci G.O. (2022). A completely digital workflow for the transition from a failed dentition to interim complete-arch fixed implant-supported prostheses: A clinical report. J. Prosthet. Dent..

[8] Papaspyridakos P., De Souza A., Bathija A., Kang K., Chochlidakis K. (2021). Complete Digital Workflow for Mandibular Full-Arch Implant Rehabilitation in 3 Appointments. J. Prosthodont..

[9] Schweiger J., Edelhoff D., Güth J.F. (2021). 3D Printing in Digital Prosthetic Dentistry: An Overview of Recent Developments in Additive Manufacturing. J. Clin. Med..

[10] Dong T., Wang X., Xia L., Yuan L., Ye N., Fang B. (2020). Accuracy of different tooth surfaces on 3D printed dental models: Orthodontic perspective. BMC Oral Health.

[11] Šimunović L., Brenko L., Marić A.J., Meštrović S., Haramina T. (2025). Rheology of Dental Photopolymers for SLA/DLP/MSLA 3D Printing. Polymers.

[12] Khaw S., Liu X., Cameron A., Aarts J., Choi J.J.E. (2023). Factors influencing the dimensional accuracy of additively manufactured dental models: A systematic review of in vitro studies. J. Mech. Behav. Biomed. Mater..

[13] Caussin E., Moussally C., Le Goff S., Fasham T., Troizier-Cheyne M., Tapie L., Dursun E., Attal J.P., François P. (2024). Vat Photopolymerization 3D Printing in Dentistry: A Comprehensive Review of Actual Popular Technologies. Materials.

[14] Tsolakis I.A., Papaioannou W., Papadopoulou E., Dalampira M., Tsolakis A.I. (2022). Comparison in Terms of Accuracy between DLP and LCD Printing Technology for Dental Model Printing. Dent. J..

[15] Alghauli M.A., Almuzaini S.A., Aljohani R., Alqutaibi A.Y. (2024). Impact of 3D printing orientation on accuracy, properties, cost, and time efficiency of additively manufactured dental models: A systematic review. BMC Oral Health.

[16] Rungrojwittayakul O., Kan J.Y., Shiozaki K., Swamidass R.S., Goodacre B.J., Goodacre C.J., Lozada J.L. (2020). Accuracy of 3D Printed Models Created by Two Technologies of Printers with Different Designs of Model Base. J. Prosthodont..

[17] Tsolakis I.A., Gizani S., Panayi N., Antonopoulos G., Tsolakis A.I. (2022). Three-Dimensional Printing Technology in Orthodontics for Dental Models: A Systematic Review. Children.

[18] Appiani A., Scattarelli P., Ori G., Noe G., Gracis S. (2024). Working Models in the Digital Workflow: Are They Reliable?. Int. J. Prosthodont..

[19] García N., Gómez-Polo M., Fernández M., Antonaya-Martín J.L., Ortega R., Gómez-Polo C., Revilla-León M., Cascos R. (2024). Influence of Printing Angulation on the Accuracy (Trueness and Precision) of the Position of Implant Analogs in 3D Models: An In Vitro Pilot Study. Appl. Sci..

[20] Rutkūnas V., Jegelevičius D., Gedrimienė A., Revilla-León M., Pletkus J., Akulauskas M., Eyüboğlu T.F., Özcan M., Auškalnis L. (2024). Effect of 3D printer, implant analog and angulation on the accuracy of analog position in implant casts. J. Dent..

